# CHIP-mediated CIB1 ubiquitination regulated epithelial–mesenchymal transition and tumor metastasis in lung adenocarcinoma

**DOI:** 10.1038/s41418-020-00635-5

**Published:** 2020-10-20

**Authors:** Yuanqi Liu, Yanwu Zhou, Pengfei Zhang, Xizhe Li, Chaojun Duan, Chunfang Zhang

**Affiliations:** 1 Department of Thoracic Surgery, Xiangya Hospital, Central South University, Changsha, 410008 PR China; 2 Hunan Engineering Research Center for Pulmonary Nodules Precise Diagnosis & Treatment, Changsha, 410008 Hunan PR China; 3 Department of Oncology, Xiangya Hospital, Central South University, Changsha, 410008 PR China; 4 National Clinical Research Center for Geriatric Disorders, Changsha, 410008 Hunan PR China

**Keywords:** Metastasis, Oncogenes, Non-small-cell lung cancer

## Abstract

CIB1 is a homolog of calmodulin that regulates cell adhesion, migration, and differentiation. It has been considered as an oncogene in many tumor cells; however, its role in lung adenocarcinoma (LAC) has not been studied. In this study, the expression levels of CIB1 in LAC tissues and adjacent normal tissues were examined by immunohistochemistry, and the relationship between CIB1 expression and patient clinicopathological characteristics was analyzed. The effects of CIB1 on epithelial–mesenchymal transition (EMT), migration, and metastasis of LAC cells were determined in vitro and vivo. Proteins interacting with CIB1 were identified using electrospray mass spectrometry (LS-MS), and CHIP was selected in the following assays. Carboxyl-terminus of Hsp70-interacting protein (CHIP) is a ubiquitin E3 ligase. We show that CHIP can degrade CIB1 via promoting polyubiquitination of CIB1 and its subsequent proteasomal degradation. Besides, lysine residue 10 and 65 of CIB1 is the ubiquitinated site of CIB1. Furthermore, CHIP-mediated CIB1 downregulation is critical for the suppression of metastasis and migration of LAC. These results indicated that CHIP-mediated CIB1 ubiquitination could regulate epithelial–mesenchymal and tumor metastasis in LAC.

## Introduction

Lung cancer is one of the most common malignant tumors and poses a severe threat to human health [1]. LAC is the most common pathological type in the past few decades, and its incidence is still proliferating [2]. LAC with poor prognosis (with about 15% 5-year survival and 30% 5-year recurrence [3]) because the mechanism of LAC is not fully understood. Therefore, it is imperative to explore the molecular mechanisms of LAC and find new therapeutic targets.

Calcium and integrin binding 1 (CIB1) is a homolog of calmodulin initially found in yeast cells [4]. CIB1 is widely expressed in systemic tissues and acts as a ligand for the platelet integrin αIIbβ3 tail cell [5]. CIB1 can promote cell proliferation, migration, and inhibition of apoptosis in various cancer by regulating several kinase partners or oncogenic signaling [6, 7]. In the early stage of this project, the LAC and its matched adjacent normal tissues were analyzed by liquid chromatography-tandem mass spectrometry and found that CIB1 was highly expressed in the LAC [8]. However, the mechanism underlying CIB1 in LAC is unknown. Since ubiquitination plays an important role in the regulation of protein expression, we sought to find the regulation of CIB1 through ubiquitinate pathway.

The degradation of the target protein by the ubiquitin-proteasome pathway requires three enzymes: ubiquitin-activating enzyme 1 (E1), ubiquitin-binding enzyme 2 (E2), and ubiquitin ligase E3 (E3). Ubiquitin is activated by E1 and transported to E2. E2 interacts directly or indirectly with a specific E3, which recognizes a specific substrate and transports the activated ubiquitin to one or more lysine residues of the substrate. The polyubiquitin chain allows the protein to be recognized by the 26S proteasome, where the protein is deubiquitinated and unfolded and eventually degraded [9]. E3 plays a crucial role in the degradation of the entire protein, determining the specificity of the reaction [10]. In this study, we found CIB1 is a novel substrate of carbonyl-terminus of hsp70-interacting protein (CHIP) which is an E3 ubiquitin ligase and closely linked with many oncogene-encoded proteins [11]. Moreover, the interaction between CIB1 and CHIP is closely linked to LAC.

## Methods and materials

### Ethics approval and consent to participate

This study was approved by the ethics committee of Xiangya Hospital, Central South University, and written informed consent was obtained from all patients (Grant number: 201703317). The Animal Ethics Committee approved all animal studies (Grant number: 2018sydw0221) and undertaken following the official recommendations of the Care and Use of Laboratory Animals of Xiangya Hospital, CSU.

### Patients and tissue specimens

A total of 60 pairs of LAC tumor tissues were obtained from patients underwent lobectomy at the Xiangya Hospital, CSU between July 2010 and December 2011. All patients were followed up every 3 months by telephone or a visit by our research team for survival and recurrence inquiry until death or until the end of the investigation.

### Cell culture and reagents

PC-9, A549, H1299, H1975, H1437, HEK293, and BEAS-2B were obtained from the Chinese Academy of Science Cell Bank (Shanghai, China). Five LAC cell lines were cultured in RPMI-1640 (12633012, Gibco, Billings, MT, USA). HEK-293 were cultured in DMEM (11965084, Gibco). BEAS-2B were cultured in BEGM Kit (CC-3170, Lonza/Clonetics Corporation). All the above mediums were supplemented with 10% FBS (10437–028, Gibco), 100 U/mL penicillin, and 100 μg/mL streptomycin (15070063, Gibco). All cell lines were passaged less than ten times after the initial revival from frozen stocks. All cell lines were authenticated prior to use by short tandem repeat profiling by Genesky Biotechnologies (Shanghai, China). AKT inhibitor MK-2206 was obtained from MCE (MK-2206, MedChemExpress, Monmouth Junction, NJ, USA).

### Plasmid and transfection

Flag-CIB1, Myc-CHIP, Myc-CHIP-ΔUbox, HA-ubiquitin plasmid were purchased from GeneChem (Shanghai, China) by cloning the corresponding human full-length DNA sequence into the 3xFlag or MYC or HA-pcDNA 3.1(+), and its mutant expression plasmid was generated by in-fusion cloning kits (TSV-S2, Tsingke, Beijing, China). Three shRNAs were constructed, and CIB1-RNAi (35732-1) was selected according to the knockdown efficiency (Supplementary Fig. S1A, Supplementary Table S1). Also, an efficient CHIP-RNAi has been selected among three constructed shRNA (Supplementary Fig. S1B, Supplementary Table S1). All mutation primers were listed in Supplementary Table S2. All transfection experiments using Lipofectamine 3000 reagents (L3000015, Invitrogen, Eugene, OR, USA) under the manufacturer’s protocols.

### Viruses and transduction

CIB1, CHIP, CHIP-ΔUbox expression, and sh-CHIP lentivirus were purchased from Vigene (Vigene, Shandong, China). The transfection methods were performed according to the manufacturer’s protocols. The transfected cells were screened by 2 μg/ml puromycin (ST551, Beyotime, Beijing, China) to obtain a stably expressed cell line.

### Immunoprecipitation and *MS*

The cell lysate for immunoprecipitation was prepared in two different methods. The cell lysate used for ubiquitin modification analysis was prepared by the denatured method. The cells plate (150 × 25 mm) were first washed by PBS and added by 0.4 ml denatured cell lysis buffer which contains 50 mM Tris (PH = 7.5) and 70 mM β-Mercaptoethanol (pre-boil for 10 min before use). Then the cells were scraped and transferred to a 2.5 ml tube, followed by 10 min boiled. The cells lysis was later added by four volumes (1.6 ml) of 1X pre-chilled cell lysis buffer (9803S, Cell signaling, Danvers, MA, USA) and be prepared for immunoprecipitation. Nondenatured cell lysis was prepared using Western &IP lysis buffer (P0013, Beyotime) containing 1X Protease inhibitor cocktail without EDTA (HY-K0010, MedChemExpress).

For co-immunoprecipitation, the supernatant was incubated with corresponding antibody overnight (2 μg antibody per 500 μg protein sample) and with protein A+G magnetic beads (HY-K0202, MedChemExpress) for 3 h at 4 °C. HA-specific magnetic beads (HY-K0201, MedChemExpress) were used for HA-fusion protein. Antibodies used in immunoprecipitation are described below: Ubiquitin (sc-8017, Santa Cruz, Dallas, TX), CIB1(11823–1-AP, Protein Tech, Chicago, IL), Flag (66008-3-Ig, Protein Tech). The magnetic beads were isolated by the magnetic racket and washed by phosphate buffered saline supplemented with 0.5% Triton-100 (PBST). Samples were eluted with 0.1 M glycine pH 3.0 adjusted to pH 7.5 with Tris buffer and run on an SDS-PAGE. After SDS-PAGE electrophoresis, proteins in the gels were detected by silver staining and followed by in-gel trypsin digestion and MS analysis as previously described by us [12, 13].

### In vitro ubiquitylation assay

2.5 μl 10× E3 Ligase Reaction Buffer (B-71, Boston Biochem), 1 μl Recombinant Human Ubiquitin Protein (U-100H-10M, Boston Biochem), 10 mM MgATP Solution (B-20, Boston Biochem), 100 nM Recombinant Human His6-Ubiquitin E1 Enzyme (E-304–05, Boston Biochem), 1 μM Recombinant Human UbcH5a/UBE2D1 Protein (E2–616–100, Boston Biochem), 1 μM Recombinant Human CHIP/STUB1 Protein (E3–220–050, Boston Biochem), 5 μM CIB1 Fusion Protein (Ag2391, Protein Tech) was added in a microcentrifuge tube and be incubated in a 37 °C water bath for 60 min. Then the lysis was added by SDS-PAGE sample buffer and followed by electrophoresis. The SDS-PAGE were then transferred onto PVDF membranes or be stained by sliver staining kits.

### Western blot

Cells were lysed using Western &IP lysis buffer (P0013, Beyotime) supplemented with 1X Protease inhibitor cocktail without EDTA (HY-K0010, MedChemExpress). Approximately 25 μg of protein extracts were separated by 10% SDS-PAGE, transferred onto PVDF membranes (ISEQ00010, Millipore). Then the PVDF membrane was blocked with 5% non-fat milk and incubated with specific antibodies. The antibodies used in WB are described below: CIB1(1:1000, ab220606, Abcam), CHIP (1:1000, ab134064, Abcam), anti-E-cadherin (1:1000, ab76055, Abcam), anti-N-cadherin (1:1000, ab18203, Abcam), anti-Vimentin (1:1000, ab92547, Abcam), anti-Ubiquitin (1:1000, 3933S, Cell signaling), anti-Tublin (1:1000, 2144, Cell signaling), anti-Flag (20543–1-AP, Protein Tech), anti-HA (66006-2-Ig, Protein Tech) and anti-Myc (60003-2-Ig, Protein Tech). An enhanced chemiluminescent (Millipore) chromogenic substrate was used to visualize the bands.

### Quantitative real-time PCR

TRIzol was used in the extraction of Total RNA. cDNA was generated using primeScript^ΤΜ^ RT reagent Kit with gDNA Eraser (RR047B, Takara, Shangdong, China). The RNA levels were quantified using SYBR Green qPCR Mix (RR820B, Takara). Primers for CIB1, CHIP, and β-actin were list in Supplementary Table S2. The expression level of according target relative to that of β-actin was defined as −ΔCT = −(CT CIB1/CHIP − CTβ-actin).

### Histology immunohistochemistry and immunofluorescence

Tumor sections were processed following standard methods and examined using routine H&E staining. For immunohistochemistry, antigen retrieval was done using standard citrate buffer protocol. The expression was graded as follows: negative (score 0), weak (score 1), moderate (score 2), and strong (score 3). Percentage scores were assigned as 1, 1–25%; 2, 26–50%; 3, 51–75%; and 4, 76–100%.

For immunofluorescence, cells were the culture in 24-Well plant and overnight. Also, antigen retrieval was done using standard protocol. The IHC and IF using the following antibodies: CIB1(1:200, ab56664, Abcam), CHIP (1:200, ab134064, Abcam), anti-E-cadherin (1:200, ab76055, Abcam), anti-N-cadherin (1:200, ab18203, Abcam), anti-Vimentin (1:200, ab92547, Abcam).

### Experimental metastasis in vivo

A549^luc^ with lentivirus (1 × 10^6^ in 100 μl PBS) infection were injected into 5-week-old nude male mice via the tail vein. Three nude mice were included in each group. Intraperitoneal injection of D-Luciferin potassium salt was used in locating and monitoring the neoplasm (PerkinElmer, Waltham, MA, USA). The mice were sacrificed at 42 days after the injection.

### Dimensional scratch assays and transwell assays

The 10 μl pipette tip was used to draw a straight line in the six-well plate with cells wholly fused. The initial and unhealed width was measured after replacing the serum-free medium. Three replicate wells were made in each group of cells, and each of the wells was randomly selected from three different visual fields for three times, and the average value was taken.

Cell (4 × 10^4^) were seeded into upper chambers purchased from BD bioscience (BD Biosciences, NJ, USA) using FBS-free medium. The lower chamber was filled with medium supplemented with 20% FBS. After co-cultured in 37 °C for 24 h, the lower surface of the upper chambers was fixated and counted.

### Statistical analysis

Data statistics in this study were statistically analyzed using SPSS 23. Comparisons between groups were analyzed by the *t*-test and *χ*^2^ test. The correlation between the expression level of CIB1 and the clinical features of LAC patients was analyzed by Spearman rank correlation analysis. The overall survival and disease-free survival curves of patients were drawn by the Kaplan–Meier method. In this study, *P* < 0.05 was set as a critical value for comparing whether there was a significant difference between the groups.

## Result

### CIB1 is frequently upregulated in LAC tissue and cell lines and associated with poor clinicopathological features and prognosis

**Fig. 1:**
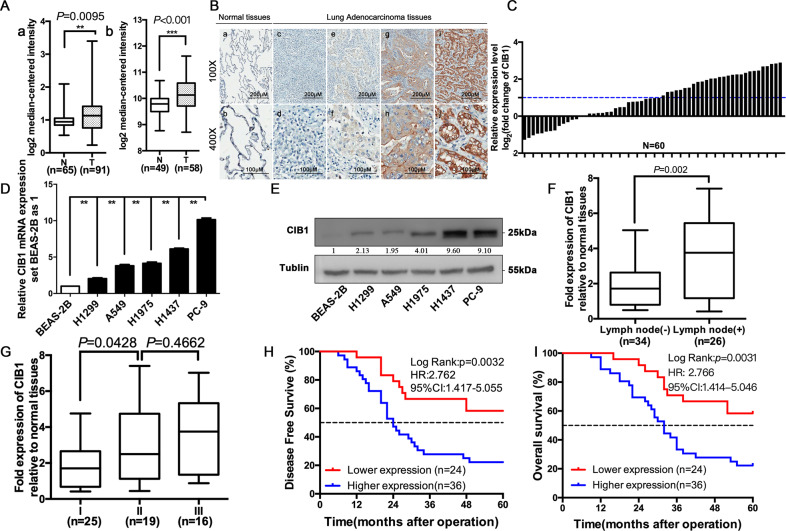
CIB1 is frequently upregulated in LAC tissue and cell lines and associated with poor clinicopathological features and prognosis. **A** Representative statistics analysis of CIB1 levels in two different data using the ONCOMINE database (www.oncomine.org). Data were filtered to display upregulation of CIB1 in LAC tissues compared to normal tissues (T: Tumor, N: normal tissues). a: Hou Lung Statistics; b: Landi Lung Statistics. **B** Representative immunohistochemically stained images of normal and LAC tissue using anti-CIB1 antibody: a, b: negative expression of CIB1 in normal lung tissue; c, d: negative expression of CIB1 in lung adenocarcinoma; e, f: weakly positive expression of CIB1 in lung adenocarcinoma; g, h: moderately positive expression of CIB1 in lung adenocarcinoma; i, j: Strong positive expression of CIB1 in lung adenocarcinoma;. **C** Relative mRNA expression level of CIB1 in LAC tumor tissue compare to normal tissues from 60 patients. **D** Quantitative RT-PCR results showed CIB1 expression levels in five LAC cell lines and normal bronchus cell lines. **E** Western blots results showed CIB1 expression levels in five LAC cell lines and normal bronchus cell lines. **F**, **G** Metastatic or advanced TNM stage LAC tumors had higher CIB1 expression levels compared with those of nonmetastatic or early TNM stage LAC tumors. **H**, **I** Kaplan–Meier analysis showed that the 5-year overall survival and disease-free survival for LAC patients with higher CIB1 expression levels were significantly shorter than those of patients with lower CIB1 expression level. *P < 0.05; **P < 0.01; ***P < 0.001; HR, hazard ratio.

In our previous study, a total of 36 differentially expressed membrane proteins were found. We then compare those identified proteins using PubMed and ONCOMINE database. CIB1 was selected as our major research object due to a higher averaged ratio-fold change (5.191 ± 0.056). Analyzes of available ONCOMING data sets from normal versus LAC tissues showed that CIB1 levels are significantly upregulated in LAC patients (Fig. 1A). The Immunohistochemical assay showed that CIB1 protein was highly expressed in LAC compared to normal tissues, mainly in the cytoplasm and cell membrane (Fig. 1B). According to the scoring criteria mentioned in the previous method, CIB1 protein was highly expressed in 60% (36/60) of LAC tissues and 30% in normal tissues, the difference was statistically significant (Supplementary Table S3). The qRT-PCR showed that the overall mRNA expression of CIB1 in LAC tissues was significantly higher than that in normal lung tissues (Fig. 1C), of which 29 (48.3%) in LAC tissues were highly expressed (higher than two times, log2 (fold change) > 1). Also, an elevated CIB1 expression was found in PC-9, H1437, H1299, H1975, and A549 cell lines compared to BEAS-2B by qRT-PCR and WB *P* < 0.01, Fig. 1D, E).

We divided the patients into two groups according to the expression level of CIB1 in tumor tissue. Clinical data of two groups are collected and compared. High expression of CIB1 was associated with N stage (*P* = 0.019), TNM stage (*P* = 0.045) (Table 1, Supplementary Table S5). What’s more, CIB1 expression was significantly higher in LAC with lymph node metastasis and advanced stage (Fig. 1F, G). Moreover, patients with high CIB1 expression in tumor tissues had shorter overall survival and disease-free survival than patients with low CIB1 expression (*P* < 0.001, Fig. 1H, I, Supplementary Fig. S2). In the COX proportional hazard regression model, the overall survival was associated with TNM stage (*P* = 0.042), CIB1 expression (*P* = 0.058) and disease-free survival were associated with TNM stage (*P* = 0.016), CIB1 expression (*P* = 0.036) (Table 2). All those were suggesting that CIB1 is highly expressed in LAC and closely associated with poor clinical characteristic and prognosis.

**Table 1:** Correlation analysis between CIB1 expression and clinical characteristics.

Variables	*n*	CIB1 expression		*P*
		Low	High	
Age (years)				0.598
≤55	30	11	19	
>55	30	13	17	
Gender				1.00
Female	25	12	18	
Male	35	12	18	
Smoking history (years)				0.752
≤10	31	13	18	
>10	29	11	18	
Tumor differentiation				0.261
High	19	8	11	
Medium	29	9	20	
Low	12	7	5	
T stage				0.831
T1	30	13	17	
T2	18	7	11	
T3	12	4	8	
N stage				0.019
N0	34	18	16	
N1–2	26	6	20	
TNM stage				0.045
I	28	15	13	
II–III	32	9	23

**Table 2:** Cox regression analysis of overall survival (OS) and disease-free survival (DFS) in 60 patients with LAC cancer.

	Univariate analysis				Multivariate analysis			
	DFS		OS		DFS		OS	
	HR (95% CI)	*P*	HR (95% CI)	*P*	HR (95% CI)	*P*	HR (95% CI)	*P*
Age	1.135 (0.600–2.146)	0.698	1.023 (0.537–1.952)	0.944	1.275 (0.615–2.642)	0.359	1.089 (0.523–2.268)	0.819
Adjuvant therapy	0.643 (0.340–1.218)	0.175	0.604 (0.316–1.154)	0.127	0.435 (0.173–1.091)	0.076	0.523 (0.207–1.324)	0.171
Gender	1.315 (0.694–2.493)	0.401	1.199 (0.628–2.289)	0.582	1.980 (0.938–4.178)	0.073	1.720 (0.824–3.592)	0.149
Tumor differentiation								
High	1		1		1		1	
Middle-low	1.116 (0.558–2.234)	0.756	1.039 (0.516–2.091)	0.916	0.680 (0.283–1.634)	0.388	0.688 (0.277–1.706)	0.419
Low	0.405 (0.132–1.243)	0.114	0.402 (0.131–1.236)	0.112	0.460 (0.126–1.676)	0.239	0.513 (0.143–1.843)	0.306
Tumor size								
T1	1		1		1			
T2	1.426 (0.674–3.020)	0.354	1.424 (0.673–3.011)	0.356	1.250 (0.491–3.182)	0.639	1.289 (0.500–3.325)	0.600
T3	2.426 (1.082–5.439)	0.031	1.927 (0.845–4.395)	0.119	1.848 (0.712–4.797)	0.207	1.440 (0.551–3.763)	0.456
Lymphatic metastasis	5.952 (2.968–11.936)	0.000	6.950 (3.384–14.273)	0.000	1.714 (0.508–5.781)	0.385	2.081 (0.578–7.483)	0.262
TNM classification								
I	1				1		1	
II	2.555 (1.098–5.944)	0.029	2.288 (0.968–5.411)	0.059	1.720 (0.634–4.668)	0.287	1.447 (0.502–4.175)	0.494
III	10.216 (4.167–25.051)	0.000	10.551 (4.171–26.688)	0.000	6.040 (1.391–26.228)	0.016	4.738 (1.054–21.296)	0.042
CIB1 expression	2.834 (1.370–5.864)	0.005	2.696 (1.299–5.596)	0.008	2.531 (1.064–6.019)	0.036	2.316 (0.973–5.513)	0.058

### CIB1 promotes LAC migratory capacity, metastatic ability, and induce EMT via AKT pathway in vitro

Clinical characteristic demonstrated that overexpression of CIB1 is positive related to tumor metastatic. Therefore, we access the ability of CIB1 in the metastatic capacity in LAC cells. Four cell lines, A549/H1299 (with lower CIB1 expression) and PC-9/H1437 (with higher CIB1 expression), were picked to perform the further study.

**Fig. 2:**
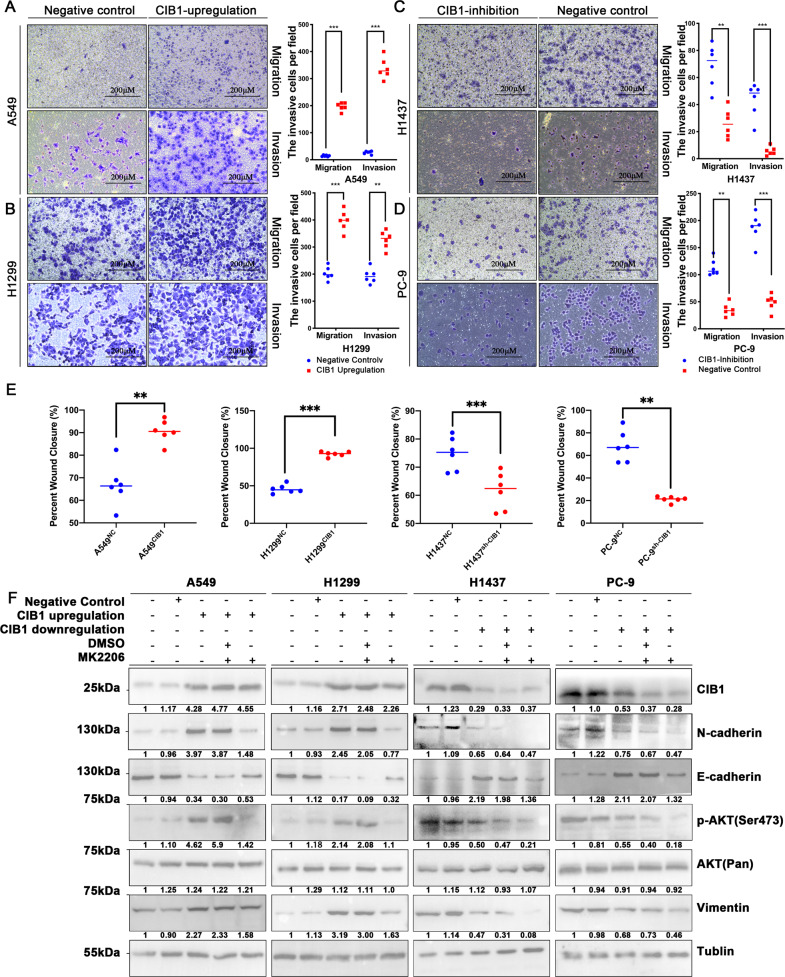
CIB1 promote LAC migratory capacity, metastatic ability, and induce EMT via AKT pathway in vitro. **A**, **B** Representative images and quantification of the Transwell assay using CIB1-upregulation A549/H1299 cells. **C**, **D** Representative images and quantification of the Transwell using anti-CIB1-transfected H1437/PC-9 cells. **E** Quantification of wound healing assays using CIB1/anti-CIB1-transfected LAC cells. **F** Western blot assay showing AKT (pan), p-AKT, E- cadherin, N-cadherin, and Vimentin protein level in CIB1/anti-CIB1-transfected LAC cells. Cells were treated with 5 μM MK2206 for 24 h. *P < 0.05 vs negative control (NC) group, **P < 0.01 vs NC group, ***P < 0.001 vs NC group.

The transwell assays and wound healing assays to evaluate the ability of LAC metastasis and migration. Overexpression of CIB1 accelerates A549 and H1299 cell migration in wound healing and transwell assay (Fig. 2A, B, E, Supplementary Fig. S3). Meanwhile, knocking down the expression of CIB1 in PC-9 and H1437 cells, the ability of according migration was also weakened in the following transwell assays and wound healing assays (Fig. 2C, D, E; Supplementary Fig. S3).

Studies have proved that overexpression of CIB1 can lead to abnormal activation of AKT, thereby promoting the development of tumor cells [6]. Since AKT pathway plays a central role in a variety of oncogenic processes including cell growth, proliferation, apoptotic cell death, motility, angiogenesis, and metastasis [14], we further test activation of AKT in transfected LAC cell. The WB result showed that in LAC cells, CIB1 could activate the AKT pathway by promoting phosphorylation of AKT in LAC (Fig. 2F). Also, activation of PI3K/AKT is a central feature of EMT development [15]. Further WB showed that the EMT marker expression was altered after CIB1 downregulation in PC-9 cells and H1437 cells, including downregulation of N-cadherin and Vimentin and upregulation of E-cadherin (Fig. 2F). When CIB1 was upregulated in H1299, and A549 cells by transfection, N-cadherin, and Vimentin expression increased, and E-cadherin expression decreased (Fig. 2F). Therefore, we hypothesize that CIB1 may promote EMT marker expression in LAC cells via PI3K/AKT pathway, contributing to the increased migration and invasion abilities that ultimately lead to the tumorigenesis. To further test this hypothesis, we subjected correspondent transfected LAC cells to the sublethal dose of MK2206, a tested AKT inhibitor. We found the compromised upregulated expression of N-cadherin and Vimentin and excessive E-cadherin protein production in CIB1 transfected H1299 and A549 cell lines after treated with MK-2206 (5 μΜ, 24 h). Further, the adverse effects were also observed in the MK2206 treated CIB1 downregulated cell lines (Fig. 2F). The results indicate that CIB1 can promote EMT via the AKT pathway in LAC cells.

### CHIP interacts with CIB1 and reduces the protein level of CIB1

**Fig. 3:**
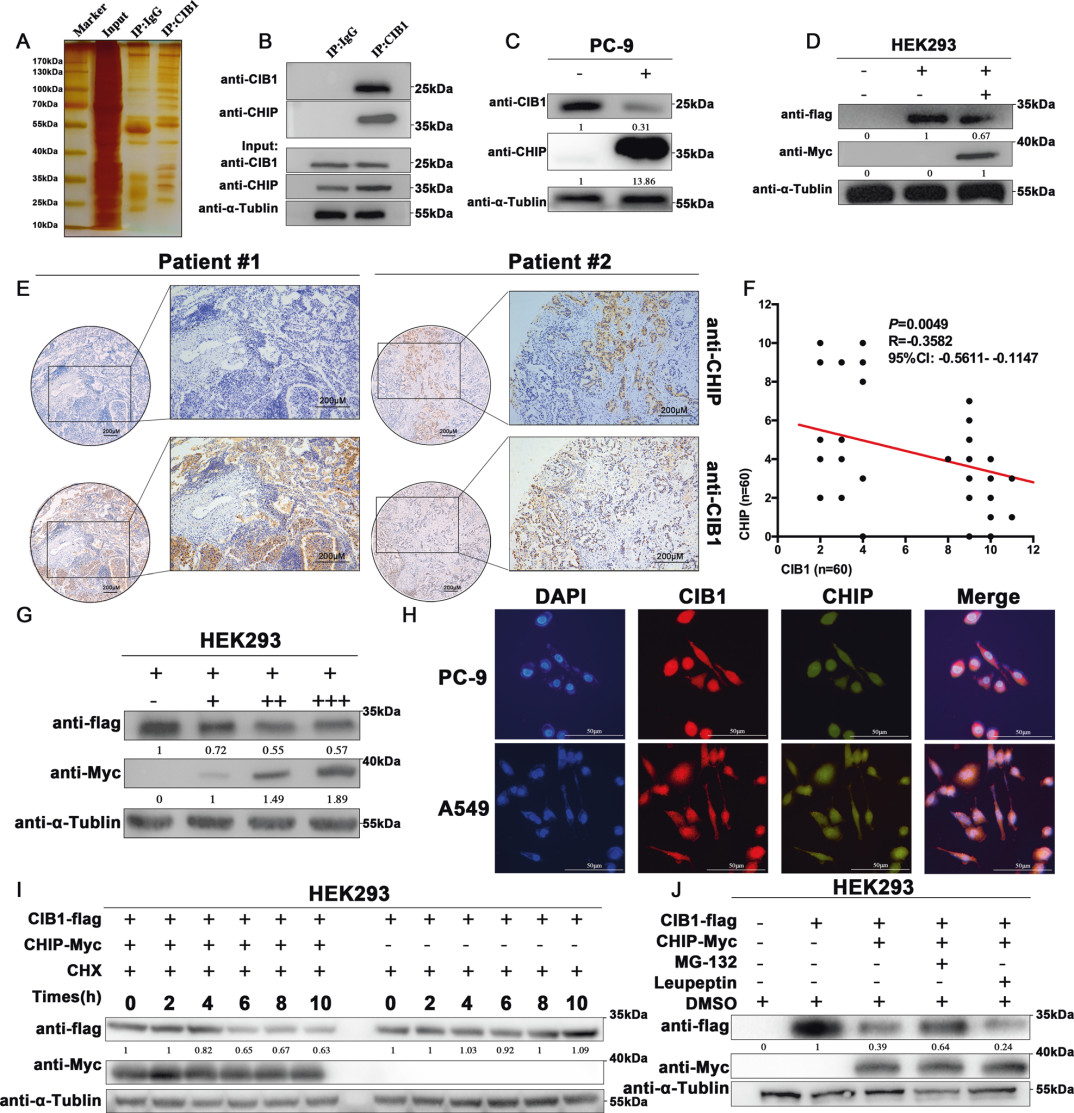
CHIP interacts with CIB1 and decreases the protein level of CIB1. CIB1 and CHIP specifically interact. **A** SDS-PAGE gel of proteins bound to IgG (left lane) or CIB1 (right lane). Protein marker stands stand for (from top to bottom): 170, 130, 100, 70 (Red), 55, 40, 35, 25, 15/10 kDa. **B** Endogenous interaction between CIB1 and CHIP in PC-9 cells. **C** PC-9 cells were transiently transfected for 36 h with CHIP plasmid, and cell lysates were immunoblotted with indicated antibodies. **D** HEK293 cells were transiently transfected for 36 h with Flag-CIB1 and Myc-CHIP, and cell lysates were immunoblotted with indicated antibodies. **E** Representative immunohistochemically stained images of LAC tissues using the anti-CHIP and anti-CIB1 antibodies. Areas in the black squares are magnified in the right slide panels. **F** Pearson analysis correlation between CIB1 and CHIP expression in LAC tissues (*r* = −0.3582, *P* = 0.0049) **G** HEK293 cells were transfected with Flag-CIB1 (1 μg) and various concentrations of Myc-CHIP plasmids (0, 0.2, 0.5, and 1 μg). And after 36 h, cell lysates were immunoblotted with anti-Flag, anti-Myc, and anti-Tublin antibodies. **H** Representative confocal images of immunostaining for CHIP (green) and CIB1 (red) in PC-9 and A549 cells. Scale bar, 50 μm. **I** Cells were treated with 40 μg/ml cycloheximide for the indicated times, and cell lysates were immunoblotted with indicated antibody. **J** HEK293 cells were transfected for 36 h with Flag-CIB1 alone or together with Myc-CHIP and treated with MG-132 (10 μM), or leupeptin (100 μM) for 4 h. Cell lysates were analyzed by western blotting with the indicated antibodies.

To identify CIB1-interacting proteins, immunoprecipitation assay using anti-CIB1 or IgG antibody followed by electrospray mass spectrometry (LS-MS) was used. The proteins met the following criteria were considered as differentially expressed proteins between the two types of immunoprecipitated: proteins were identified based on ≥2 peptides with ≥95% confidence and showed an averaged ratio-fold change ≥ 1.5 (the student’s *t* test, *P* < 0.05) in the two LC-MS analyses. According to these criteria, a total of 38 differentially expressed proteins were found (Fig. 3A, Supplementary Table S4). Since ubiquitin-proteasome systems closely link to the degradation of protein, we choose CHIP, the only E3 ubiquitin ligase being identified, for the following assays.

In PC-9 cell lines, immunoprecipitated using anti-CIB1 antibody followed by WB using anti-CHIP antibody showed the combination between CIB1 and CHIP (Fig. 3B). Immunofluorescence using CIB1 and CHIP antibodies in PC-9 and A549 cell lines showed that two proteins were co-expressed in the cell both in the cytoplasm and nucleus (Fig. 3H).

After observing the interaction between CIB1 and CHIP, we then found the suppressed endogenous and exogenously CIB1 was also observed in the CHIP overexpressing PC-9 and HEK293 cell lines (Fig. 3C, D). Moreover, in the HEK293 cell, we observed that CHIP could decrease CIB1 in a dose-dependent manner (Fig. 3G). Further immunohistochemical staining of 60 patients showed that CHIP is lowly expressed in tissues and is significantly negatively correlated with CIB1 expression (Fig. 3E, F). RT-qPCR results show that the mRNA expression levels of CIB1 in CHIP overexpressing PC-9 did not show any difference, indicating that CHIP regulates the stability of CIB1 protein in the post-transcriptional levels (Supplementary Fig. S4). We used CHX to measure the half-life of CIB1, showing that CHIP attenuates CIB1 stability (Fig. 3I). Overexpression of Myc-CHIP can downregulate the protein level of CIB1, but after adding MG-132 (proteasome inhibitor), the protein level of CIB1 downregulated by CHIP is reversed. However, and the protein level of CIB1 down regulated by CHIP was not reversed after treating with leupeptin (Fig. 3J). All those were indicating that CHIP-induced decline in CIB1 protein levels is dependent on the proteasome pathway.

### CHIP facilitates K48-linked polyubiquitination of CIB1

**Fig. 4:**
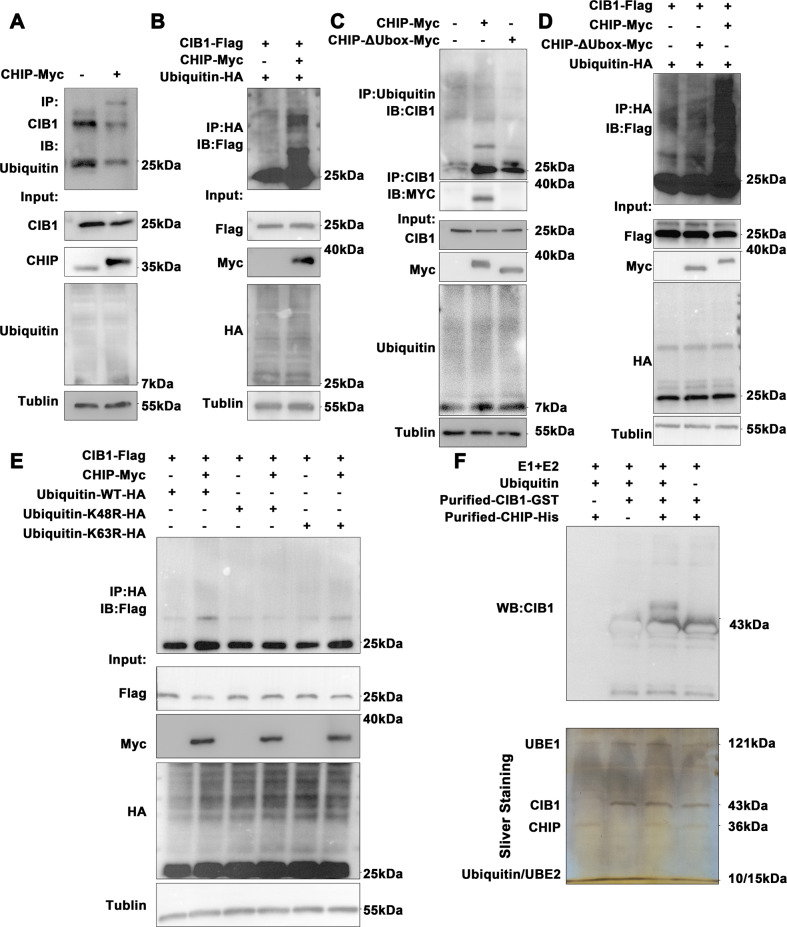
CHIP facilitates K48-linked polyubiquitination of CIB1. **A** PC-9 were transfected with CHIP plasmid for 36 h. Cell extracts were immunoprecipitated with the anti-CIB1 antibody, followed by immunoblotting with the anti-Ubiquitin antibody. **B** HEK293 cells were transfected with HA-ubiquitin, Flag-CIB1 with or without Myc-CHIP. Cell lysates were immunoprecipitated with the anti-HA antibody, followed by immunoblotting with anti-flag antibodies. **C** PC-9 cells were transfected with CHIP or CHIP-ΔUbox plasmid. Cell extracts were immunoprecipitated with the anti-ubiquitin antibody, followed by immunoblotting with the anti-CIB1 antibody. **D** HEK293 cell were transfected with Flag-CIB1, HA-ubiquitin, with or without Myc-CHIP or Myc -CHIP-ΔUbox. Denatured cell lysates were immunoprecipitated with the anti-HA antibody, followed by immunoblotting with Flag antibodies. **E** Where specified, purified E1, E2, ubiquitin, CHIP and CIB1 proteins were incubated with in vitro ubiquitination buffers. Reaction samples were analyzed by SDS-polyacrylamide gel electrophoresis, followed by Sliver staining or immunoblotting with the indicated antibodies. **F** HEK293 cells were transfected for 36 h with Flag-CIB1, Myc-CHIP, HA-Ubiquitin or HA-K48R-ubiquitin or HA-K63R-ubiquitin. Cell extracts were immunoprecipitated with the anti-HA antibody followed by immunoblotting with the anti-Flag antibody.

After demonstrating that CHIP may promote CIB1 degradation through ubiquitination-proteasome pathways. We overexpressed CHIP and blank plasmids in PC-9 cells, followed by using CIB1 antibody to precipitate CIB1 protein and detected it with ubiquitin antibody. WB results showed that the level of ubiquitinated CIB1 is significantly higher in cells transfected with CHIP than in cells transfected with blank plasmid alone (Fig. 4A). Moreover, when flag-CIB1, HA-ubiquitin, and Myc-CHIP expression plasmid were co-transfected into HEK293T cells, the level of ubiquitination of flag-CIB1 was significantly increased (Fig. 4B). The U-box domain constitutes a loop finger structure (RNG-finger), which is a domain that functions as a ubiquitin ligase. To compare the ubiquitination of CIB1 by CHIP, we constructed the CHIP-ΔUbox plasmid by deleting the Ubox domain of CHIP. We found that CHIP-ΔUbox mutants could not increase ubiquitination levels of CIB1, both endogenously and exogenously (Fig. 4C, D). Moreover, in vitro ubiquitination assays with recombinant CHIP and CIB1 showed that CHIP markedly enhances CIB1 ubiquitination (Fig. 4F), suggesting that CHIP directly ubiquitinates CIB1. Subsequently, we transferred K48 and K63 into HEK293 cells, respectively. The CHIP-mediated ubiquitination of CIB1 disappeared after the 48th lysine mutation on ubiquitin, while the K63 mutation did not change (Fig. 4E). All those were suggesting that CHIP promotes CIB1 ubiquitination via the Lys-48 residue of ubiquitin.

### CHIP targets CIB1 lysine 10 and lysine 65 for ubiquitination

**Fig. 5:**
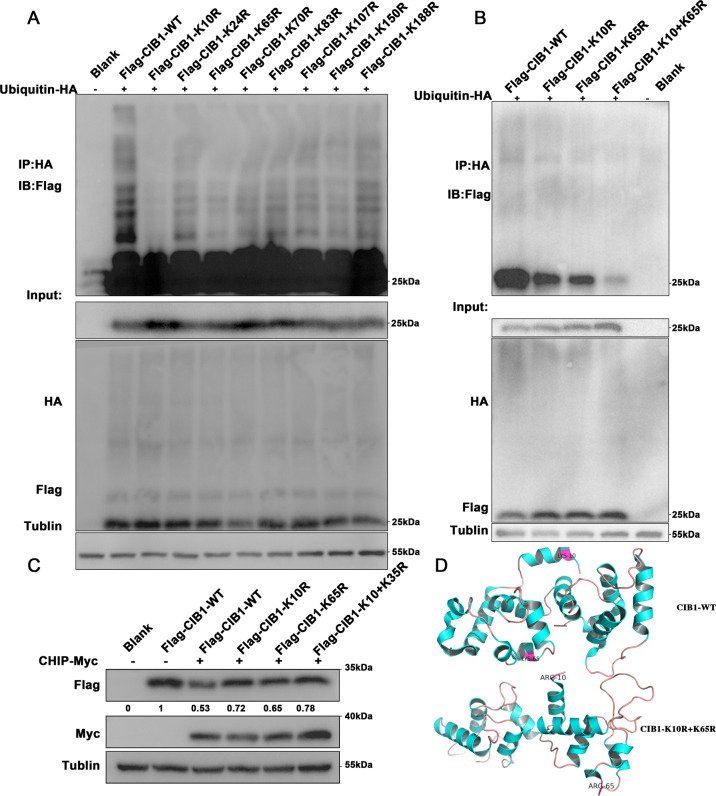
Multiple K residues of CIB1 are polyubiquitinated by CHIP. **A** HEK293 cells were co-transfected with HA-ubiquitin and Flag-CIB1 and its corresponding mutated plasmids for 36 h. Cell lysates were subjected to immunoprecipitation with HA antibody, followed by Flag immunoblotting. **B** HEK293 cells were co-transfected with HA-ubiquitin with HA-ubiquitin, and Flag-CIB1 or Flag-K10R-CIB1 or Flag-K65R-CIB1 or Flag-K10R+K65R-CIB1. Cell lysates were subjected to immunoprecipitation with HA antibody, followed by Flag immunoblotting. **C** HEK293 cells were co-transfected with Flag-CIB1, Flag-K10R-CIB1, Flag-K65R-CIB1, Flag-K10R+K65R-CIB1 and Myc-CHIP. Cell lysates were analyzed by immunoblotting with MYC, Flag and α-Tublin antibodies. **D** Computational modeling results show multiple binding sites for ubiquitin on CIB1. The red region is the binding site for CIB1.

Since ubiquitination modification usually occurs in the lysine of a protein substrate, we further sought to explore which lysine on CIB1 may be the sites that cause ubiquitination modification. Eight lysine residues on the CIB1, K10, K24, K65, K70, K83, K107, K150, K188 were mutated to ornithine (Supplementary Fig. S5), and co-transfected into HEK293 cells combine with HA-ubiquitin. Results showed that the ubiquitinated CIB1 was significantly decreased after lysine 10, and lysine 65 was mutated (Fig. 5A). Therefore, we speculate that lysine 10 and lysine 65 may be ubiquitination sites in CIB1 protein. It was further found that after the simultaneous mutation of K10 and K65, the ubiquitination of CIB1 showed a significant decrease compared with the individual mutations (Fig. 5B). Also, the Flag-CIB1 downregulation caused by CHIP overexpression was retrieved when K10 and K65 were co-mutated to ornithine (Fig. 5C). All those were suggesting that lysine 10 and lysine 65 may be the ubiquitinated site of CIB1 (Fig. 5D).

### CIB1 is negatively regulated by CHIP and affects the metastatic ability of lung adenocarcinoma cells in vitro

**Fig. 6:**
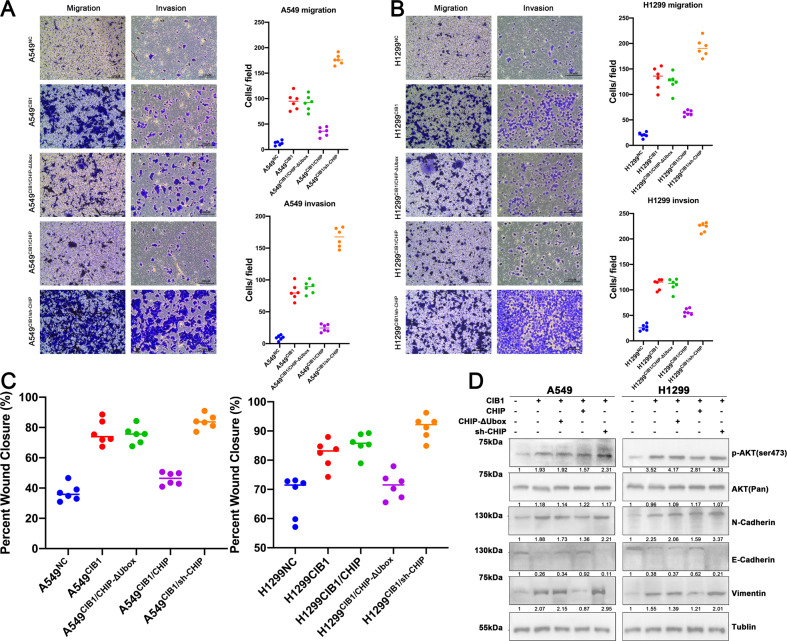
CHIP upregulation alleviates CIB1 induced LAC migratory capacity and metastatic ability in vitro. **A**, **B** Representative images and quantification of the Transwell assays using transfected A549/H1299 cells. **C**, **D** Quantification of the wound healing assays using transfected A549/H1299 cells. **E** Western blot assay showing the phosphorylation of AKT and EMT enhanced by CIB1. Restoration of CHIP attenuated this elevation in LAC cells.

After identifying the interaction between CIB1 and CHIP, we further examined the effectiveness of CHIP on the CIB1 mediated enhanced LAC migration and invasion in vitro. In A549 and H1299 cell lines, we simultaneously overexpressed both CIB1 and CHIP proteins using the corresponding plasmid. The transwell assays and wound healing assays were used to evaluate the invasion and migration ability. The results showed that the CIB1 induced LAC invasion, and metastasis of LAC was subdued by over-expressed CHIP, but CHIP-ΔUbox did not produce the same effects (Fig. 6A–C, Supplementary Fig. S6). In additional, knocking down CHIP expression by its shRNA significantly enhanced LACs’ migratory ability under CIB1-overexpression conditions, indicating an enhanced cell migration was promoted by CHIP downregulation (Fig. 6A–C, Supplementary Fig. S6). Moreover, WB shows that p-AKT showed a significant turnover when CIB1 and CHIP were co-transfections in H1299 and A549 cell lines (Fig. 6D). Further, we exam the EMT marker (Vimentin, E-Cadherin, N-Cadherin) in those two transfected cells lines. The WB results show that the elevated N-Cadherin and Vimentin induced by CIB1 and reversed downregulated by CHIP overexpression, whereas the CHIP-ΔUbox cannot have the same results. Moreover, the inhibition of E-cadherin caused by CIB1 overexpression can be overturned by CHIP upregulation but not through CHIP-ΔUbox (Fig. 6E). However, transfected-mediated expression of CHIP shRNA significantly boosted the CIB1-induced upregulation of N-cadherin and Vimentin protein, reversed that of E-cadherin protein (Fig. 6E). All those indicated that CIB1 is negatively regulated by CHIP and affects the metastatic ability of lung adenocarcinoma cells in vitro.

### CIB1 is negatively regulated by CHIP and affects the metastatic ability of lung adenocarcinoma cells in vivo

**Fig. 7:**
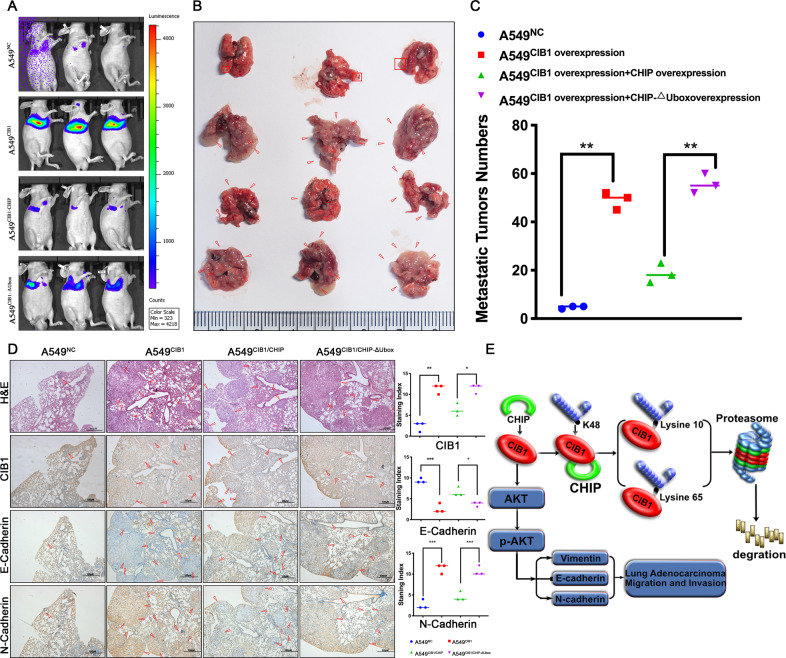
CHIP upregulation alleviates CIB1 induced LAC migratory capacity and metastatic ability in vivo. **A** Representative luciferase images and quantification of average luciferase intensity of lungs in the i.v. metastasis assay. **B**, **C** Representative photographs and quantification of metastatic tumor nodes in mouse lungs from the i.v. metastasis assay. **D** Representative immunohistochemically stained images of lung tissue using anti-CIB1, anti-E-cadherin, and N-cadherin antibody. **E** CHIP-mediated CIB1 ubiquitination regulated epithelial–mesenchymal and tumor metastasis in lung adenocarcinoma through AKT pathway. *P < 0.05 vs negative control (NC) group, **P < 0.01 vs NC group.

After found that CHIP can alleviate CIB1 induced LAC enhanced migratory capacity and metastasis ability in vitro, we further examine whether the same result can be observed in vivo. A549^NC^ and A549^CIB1^ cells were injected into the tail vein of nude mice. Also, A549^CIB1/CHIP^ and A549^CIB1/ΔUbox^ were injected according to previously described methods. All the mice underwent bioluminescence imaging and were sacrificed for the subsequent assays after 42 days. Compared to the NC, A549^CIB1^ injected mice had enhanced luminescence intensity. Also, A549^CIB1/CHIP^ have lower node compare to A549^CIB1/CHIP-ΔUbox^ (Fig. 7A–C). The same result was also observed in HE stains result. Moreover, IHC using N-cadherin and E-cadherin show corresponding alternation (Fig. 7D). Yet, another group of nude mice was sacrificed to test whether downregulating CHIP expression could greatly enhance the CIB1-overexpressed LAC migratory capacity and metastasis ability. It was notable that at 42 days following the tail vein rejection, an elevated metastasis node was observed in mice lung (Supplementary Fig. S7). Thus, we proved that CIB1 can negatively be regulated by CHIP and affects the metastatic ability of lung adenocarcinoma cells in vivo.

## Discussion

CIB1 was initially thought to be a ligand for the platelet integrin αIIbβ3 tail cell [4] and a small intracellular protein that regulates kinase and integrin activity [5, 16–18]. CIB1 has been shown influenced many tumor cells activity via multiple signaling pathways [7, 19, 20]. In this study, based on previous reviews in our laboratory, we first explored the expression and clinical significance of CIB1 in LAC [8]. We found that CHIP can interact with CIB1 and degraded CIB1 through the ubiquitin-proteasome pathway. The interaction of CIB1 and CHIP then controlled the invasion and migration of lung adenocarcinoma.

We first showed that CIB1 is highly expressed in LAC and associated with adverse clinical feature and prognosis. Similar results were observed in hepatic carcinoma and triple-negative breast cancer [6, 7]. After finding that the expression level of CIB1 is related to LAC metastasis, we further sought to evaluate the ability of CIB1 in regulating the invasion and migration of LAC cells. The wound healing assays and transwell assays showed that LAC cell lines with CIB1 could significantly affect invasive and migration ability. AKT is a serine/threonine-protein kinase that transmits extracellular stimuli and involved in a series of physiological activities such as apoptosis, proliferation, and differentiation [21]. The PI3K/AKT signaling pathway also is closely related to the development, invasion, and metastasis of human tumors [6, 22]. This study found that overexpression of CIB1 in LAC could also significantly promote AKT activation. Studies have found that abnormal activation of NF-κB, PI3K/Akt, Notch, Wnt/β-catenin, TGF-β, integrin, JAK/STAT3, and Hedgehog can lead to EMT [23–26]. Epithelial–mesenchymal transition (EMT) refers to the morphological changes of epithelial cells to stromal cells or fibroblasts. Abnormal activation of EMT plays an essential role in the development of LAC [27–29]. The previous report has found that Integrin, a binding protein of CIB1, plays an important role in the development of lung cancer by participating in EMT [26]. In the advanced non-small cell lung cancer, silencing E-cadherin can promote the transformation of cells into EMT, while the restoration of E-cadherin expression can actively restore the invasion or migration of tumor cells [30]. While N-cadherin and Vimentin act as mesenchymal cell expression molecules and upregulation of their expression promotes metastasis of LAC [31, 32]. We found that a marked change in the expression of Vimentin, N-cadherin, and E-cadherin when CIB1 expression change. It indicates that CIB1 may promote EMT in LAC cells by activating the PI3K/AKT signaling pathway.

To find proteins that interact with CIB1, we used electrospray mass spectrometry finding that CHIP proteins can interact with CIB1. CHIP expresses as E3 ubiquitinated ligase, which targets many mature proteins for ubiquitination and proteasomal degradation [33, 34]. CHIP has three functional domains, in which the Ubox domain in the C-terminus provided its E3 ligase activity, which makes CHIP participate in the ubiquitination and degradation process of proteins and plays a broad and important role in the biological process of cells [11, 35, 36]. Many tumor-associated proteins are substrates for CHIP: C-Myc, Ron, ASK1, ErbB2/her2, p53, ASK, etc. [37–43]. Studies have shown that CHIP can negatively regulate the vascular endothelial growth factor pathway, inhibit angiogenic ability, and thus inhibit distant metastasis of tumors [44, 45]. Also, CHIP can selectively regulate the epidermal growth factor receptor of LAC mutants through ubiquitination, thereby affecting the efficacy of chemotherapy [46]. Here we uncover that CIB1 is a new substrate for CHIP. A negative correlation between the expression of CHIP and CIB1 can be found both in cells and tissues. CHIP can affect the stability of CIB1. Notably, by using proteasomal (MG132) showed that CHIP-dependent destabilization of CIB1 primarily occurs in the proteasome. Then, the WB followed by co-precipitation show an increasing ubiquitinated CIB1 after CHIP overexpression. However, the CHIP- ΔUbox which lose the E3 ligase activity lose the ability to decrease CIB1 expression through facilitated the ubiquitination of CIB1.

Since Lysine (K) residues are ubiquitin acceptor sites for ubiquitin [47], we next sought to find the possible Lysine for CIB1 ubiquitination. In this article, we found that K10 and K65 were potential targets for CIB1 ubiquitinate bind site. After K10 and K65 were co-mutation in CIB1, the ubiquitinated CIB1 was decreased.

After finding that CHIP facilitated CIB1 degradation, we then try to test and verify whether CHIP upregulation can alleviate CIB1 induced LAC migratory capacity and metastatic ability. Previous researchers have found that CHIP was expressed in various tumors [33, 44]. In breast cancer, ovarian carcinoma, colorectal cancer, and renal carcinoma, CHIP appears to function as a suppressor of tumorigenesis [48–51]. Here, we found that the regulation of CHIP expression was proved to attenuate the ability of migration and invasion caused by CIB1 in LAC cells both in vivo and vitro. Moreover, The EMT markers, including Vimentin, E-cadherin, N-cadherin, also change with CHIP overexpression. Also, the phosphorylation of AKT was significantly repressed after CHIP transfection. In accordance with our finding, Zhang et al. have demonstrated that CHIP have the function of suppression of lung cancer metastasis [52]. Taken together, our results indicated that CHIP could suppress the EMT process in LAC cells by targeting CIB1 through the AKT pathway.

In summary, our results showed that CIB1 was significantly increased in LAC and correlated with poor prognosis of LAC patients. CIB1 could promote cell invasion and migration both in vitro and in vivo. CHIP could degrade CIB1 by facilitated CIB1 polyubiquitination of CIB1. K10 and K65 were the Lysine (K) residues for CHIP mediated CIB1 degradation. In conclusion, we have carried out research from molecular, cellular, animal models, and clinical specimens to elucidate the molecular mechanism of the interaction between CIB1 and CHIP in inducing migration and invasion of lung adenocarcinoma cells, and to provide experimental evidence for the treatment of new targets for lung adenocarcinoma. Also, we provide a theoretical basis and innovative ideas for the possible construction of a possible treatment strategy for lung adenocarcinoma of CIB1.

## Supplementary information

Supplement Fig. 1

Supplement Fig. 2

Supplement Fig. 3

Supplement Fig. 4

Supplement Fig. 5

Supplement Fig. 6

Supplement Fig. 7

Supplement Figure legends

Supplement Table 1

Supplement Table 2

Supplement Table 3

Supplement Table 4

Supplement Table 5
