# Supplementary figures and images for: CHIP-mediated CIB1 ubiquitination regulated epithelial–mesenchymal transition and tumor metastasis in lung adenocarcinoma
Source: Cell Death Differ. 2020 Oct 20;28(3):1026–40. doi: 10.1038/s41418-020-00635-5 (PMC7937682; doi:10.1038/s41418-020-00635-5)

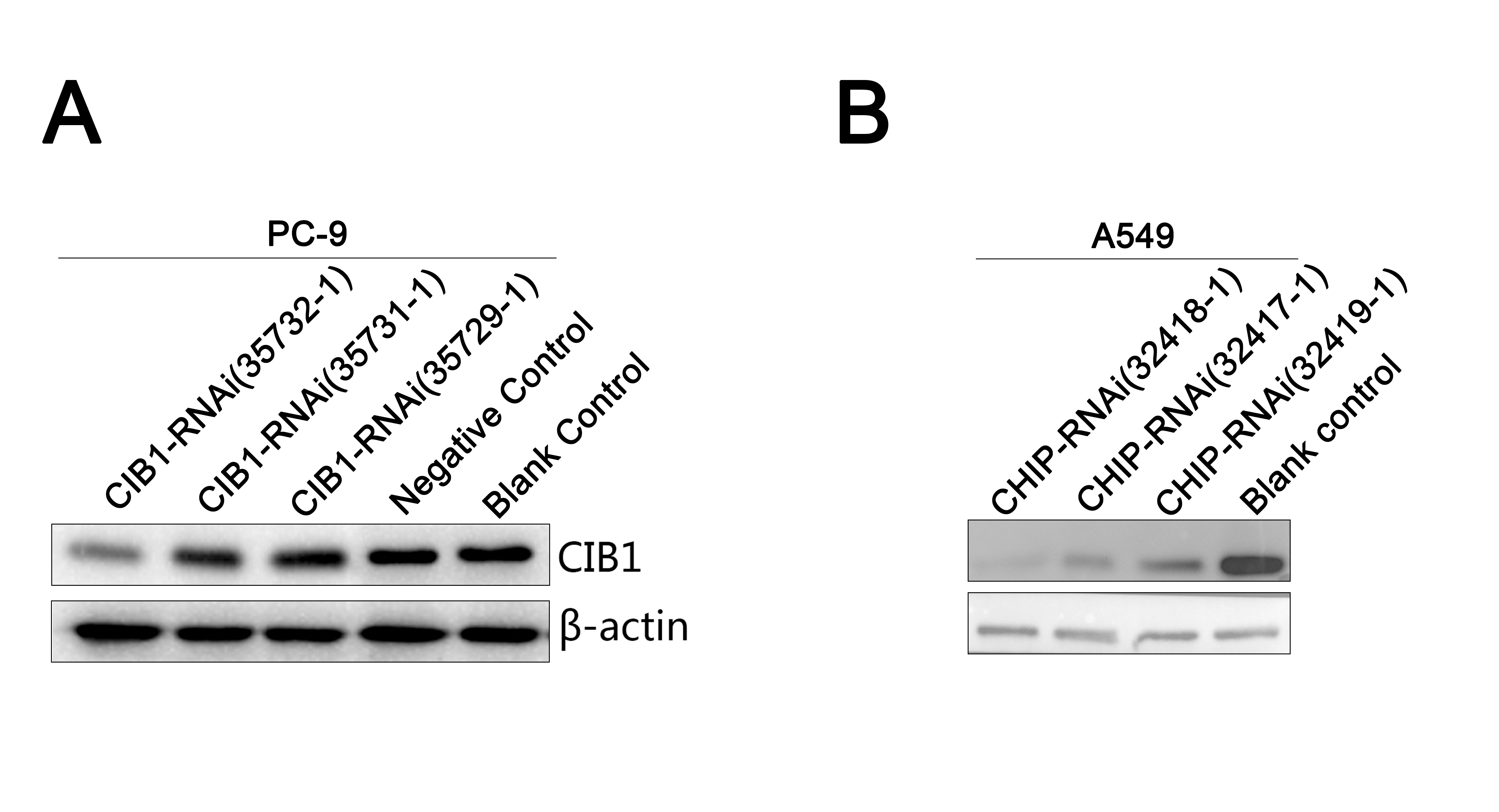

Supplement: Supplementary file 1 — Supplement Fig. 1 [file 41418_2020_635_MOESM1_ESM.png]

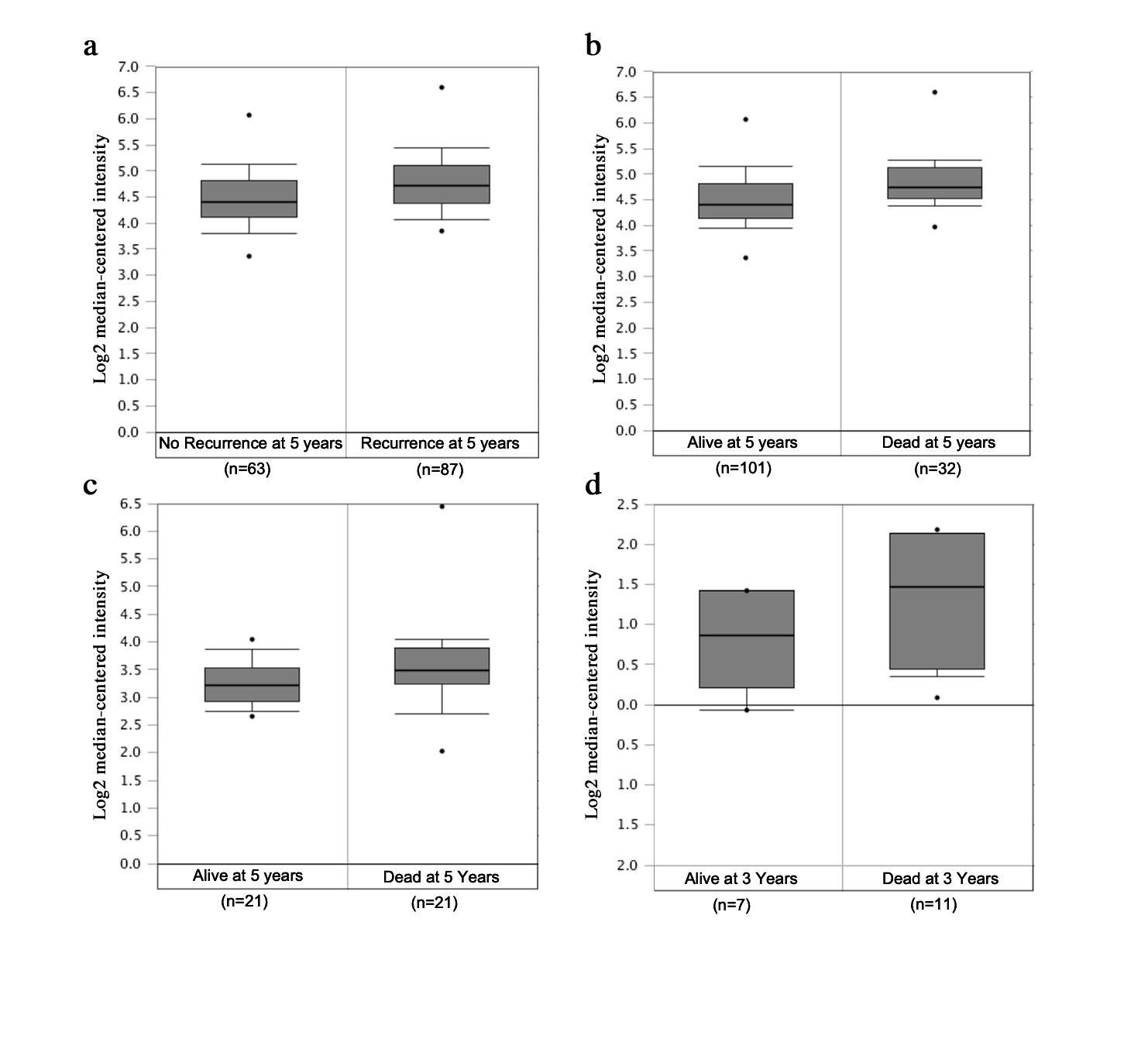

Supplement: Supplementary file 2 — Supplement Fig. 2 [file 41418_2020_635_MOESM2_ESM.png]

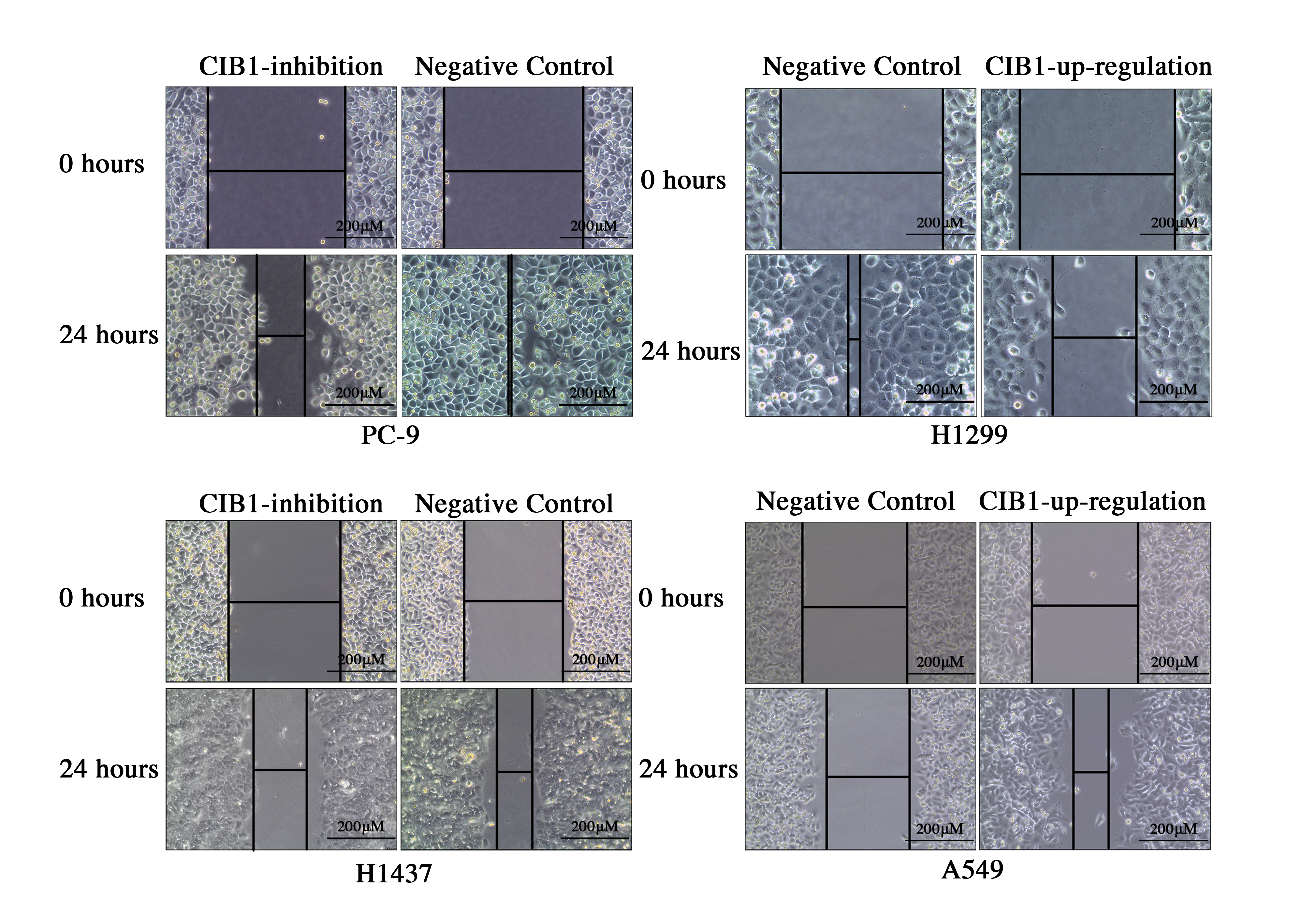

Supplement: Supplementary file 3 — Supplement Fig. 3 [file 41418_2020_635_MOESM3_ESM.png]

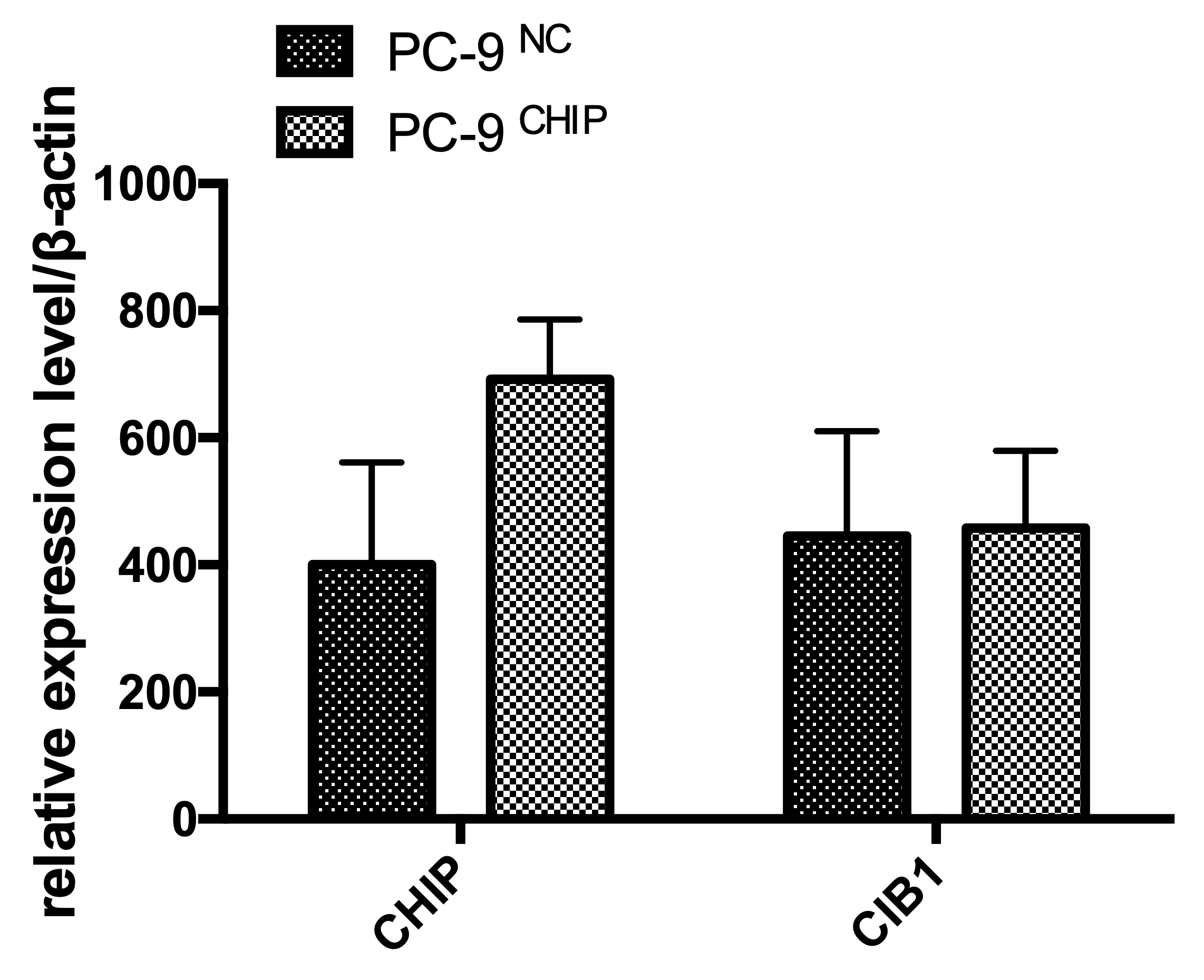

Supplement: Supplementary file 4 — Supplement Fig. 4 [file 41418_2020_635_MOESM4_ESM.tif]

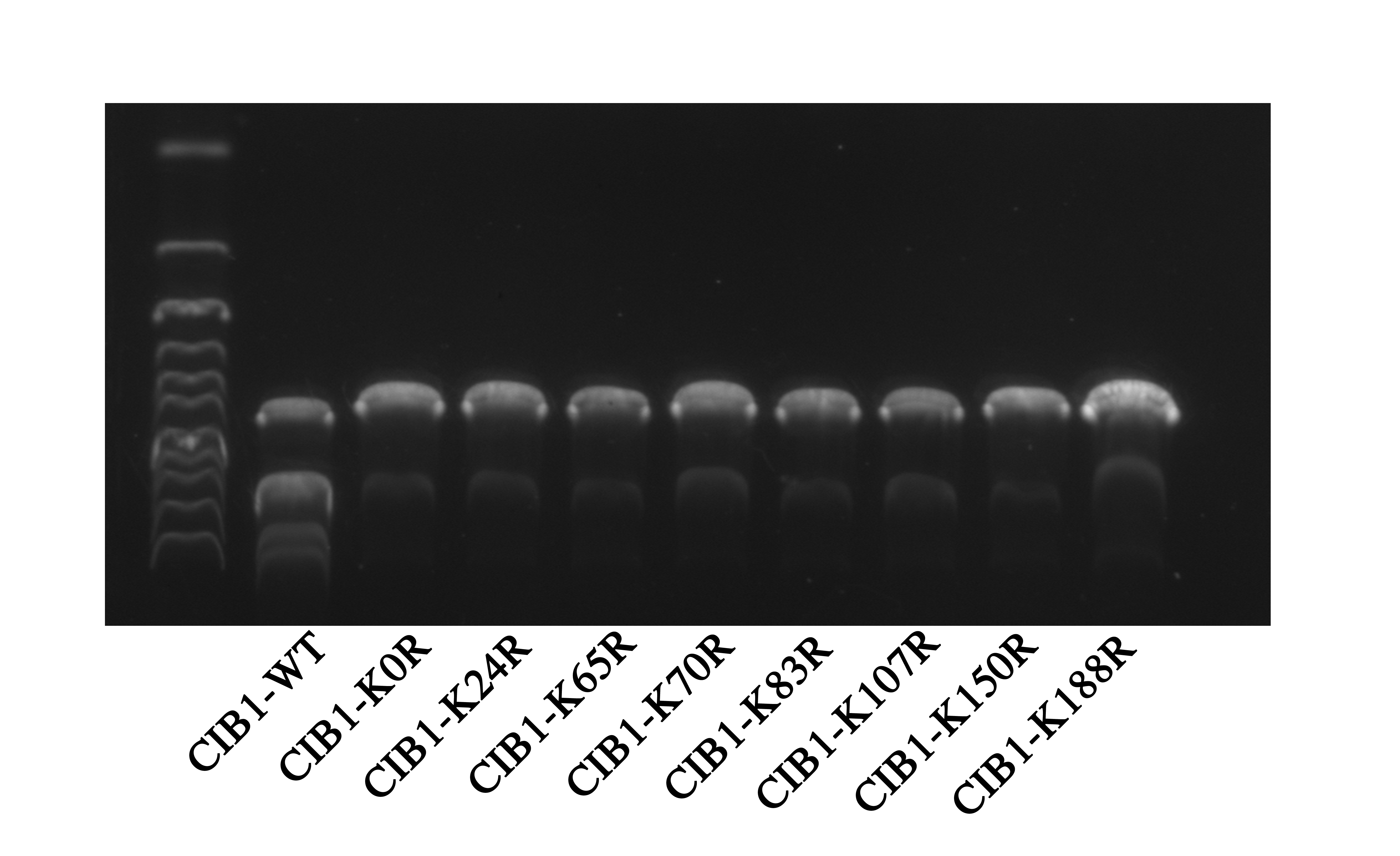

Supplement: Supplementary file 5 — Supplement Fig. 5 [file 41418_2020_635_MOESM5_ESM.png]

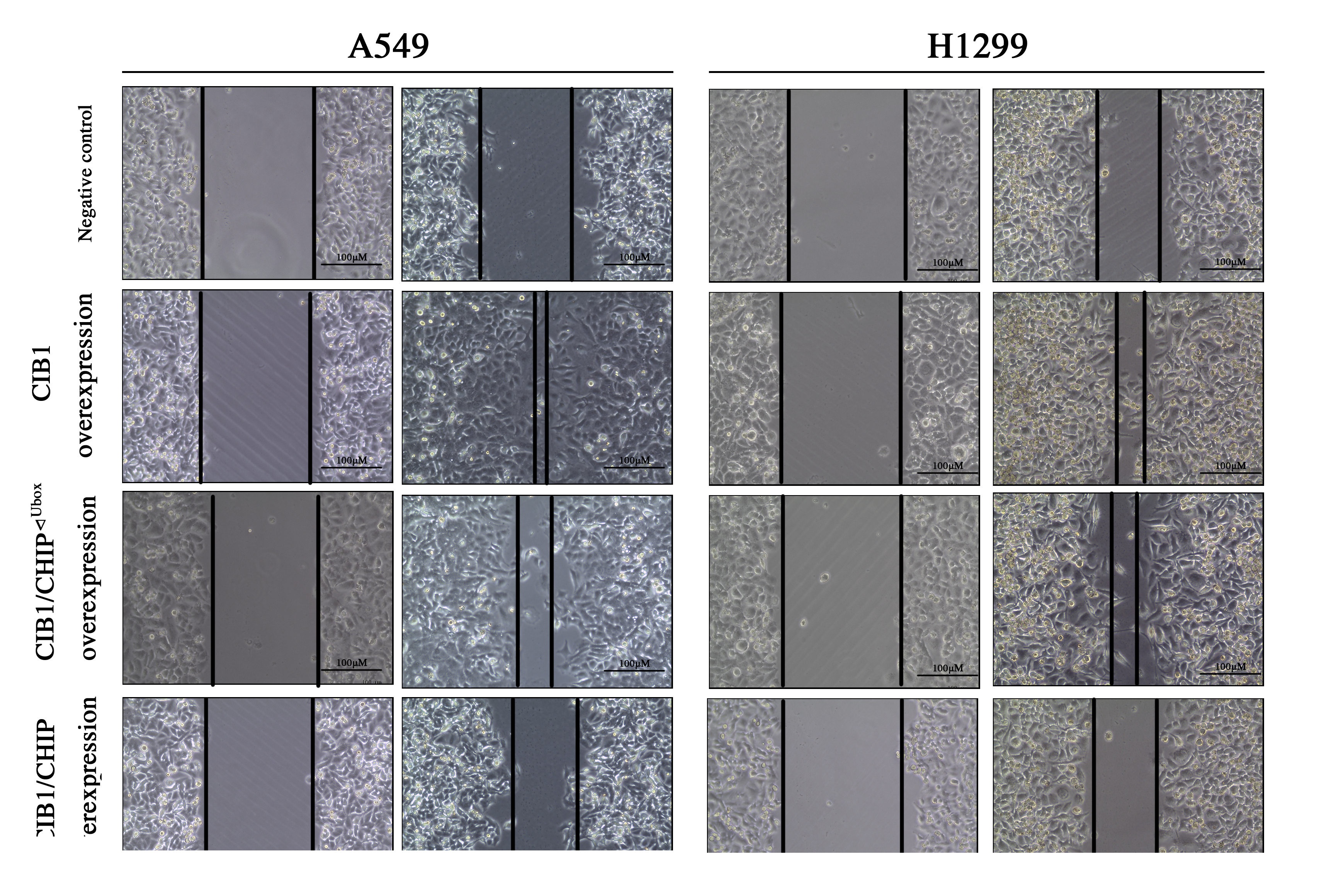

Supplement: Supplementary file 6 — Supplement Fig. 6 [file 41418_2020_635_MOESM6_ESM.png]
