# Supplementary material for: CHIP-mediated CIB1 ubiquitination regulated epithelial–mesenchymal transition and tumor metastasis in lung adenocarcinoma
Source: Cell Death Differ. 2020 Oct 20;28(3):1026–40. doi: 10.1038/s41418-020-00635-5 (PMC7937682; doi:10.1038/s41418-020-00635-5)
Supplement: Supplementary file 8 — Supplement Figure legends [file 41418_2020_635_MOESM8_ESM.docx]

Supplement figure 1. A. CIB1 down-regulates target screening. a: Western blotting method was used to detect the expression of CIB1 protein in PC-9 cells infected with CIB1-RNAi-35732, CIB1-RNAi-35731 and CIB1-RNAi-35729. B. CHIP down-regulates target screening. a: Western blotting method was used to detect the expression of CHIP protein in A549 cells infected with CHIP-RNAi-32418, CHIP-RNAi-32417 and CHIP-RNAi-32419.

Supplement figure 2. Representative statistics analysis of DFS and OS in two different data using the ONCOMINE database ([www.oncomine.org](http://www.oncomine.org)).

Supplement figure 3. Representative images of wound healing assays using CIB1/anti-CIB1-transfected LAC cells.

Supplement figure 4. Total RNA was extracted from CHIP plasmid-transfected PC-9 cells, CIB1 and CHIP mRNA levels were then examined by RT-real time PCR with CIB1 and CHIP primers. All the blots are representative of three independent experiments.

Supplement figure 5. Agarose gel electrophoresis of CIB1 and its mutant plasmid. DNA marker stands stand for (from top to bottom): 500bp, 1000bp, 2000bp, 3000bp, 4000bp, 5000bp, 6000bp, 7000bp, 8000bp, 9000bp, 10000bp.

Supplement figure 6. Representative images of wound healing assays using transfected A549/H1299 cells.

Supplement figure 7. (A) Representative luciferase images and quantification of average luciferase intensity of lungs in the i.v. metastasis assay. (B-C) Representative photographs and quantification of metastatic tumor nodes in mouse lungs from the i.v. metastasis assay. (D) Representative immunohistochemically stained images of lung tissue using anti-CIB1, anti-E-cadherin and N-cadherin antibody.
