# Supplementary material for: CHIP-mediated CIB1 ubiquitination regulated epithelial–mesenchymal transition and tumor metastasis in lung adenocarcinoma
Source: Cell Death Differ. 2020 Oct 20;28(3):1026–40. doi: 10.1038/s41418-020-00635-5 (PMC7937682; doi:10.1038/s41418-020-00635-5)
Supplement: Supplementary file 9 — Supplement Table 1 [file 41418_2020_635_MOESM9_ESM.docx]

| Supplement Table 1. Three shRNA sequence information for the CIB1 gene | | | | | |
| --- | --- | --- | --- | --- | --- |
| NO. | 5’ | STEM | Loop | STEM | 3’ |
| CIB1-RNAi(35729-1)-a | Ccgg | ccAGACTTTGCCAGCTCCTTT | CTCGAG | AAAGGAGCTGGCAAAGTCTGG | TTTTTg |
| CIB1-RNAi(35729-1)-b | aattcaaaaa | ccAGACTTTGCCAGCTCCTTT | CTCGAG | AAAGGAGCTGGCAAAGTCTGG |  |
| CIB1-RNAi(35731-1)-a | Ccgg | ccAGACATCAAGTCCCATTAT | CTCGAG | ATAATGGGACTTGATGTCTGG | TTTTTg |
| CIB1-RNAi(35731-1)-b | aattcaaaaa | ccAGACATCAAGTCCCATTAT | CTCGAG | ATAATGGGACTTGATGTCTGG |  |
| CIB1-RNAi(35732-1)-a | Ccgg | caGCCTTAGCTTTGAGGACTT | CTCGAG | AAGTCCTCAAAGCTAAGGCTG | TTTTTg |
| CIB1-RNAi(35732-1)-b | aattcaaaaa | caGCCTTAGCTTTGAGGACTT | CTCGAG | AAGTCCTCAAAGCTAAGGCTG |  |
| STUB1-RNAi(32417-1)-a | Ccgg | gaAGAGGAAGAAGCGAGACAT | CTCGAG | ATGTCTCGCTTCTTCCTCTTC | TTTTTg |
| STUB1-RNAi(32417-1)-b | GATCCAAAAA | gaAGAGGAAGAAGCGAGACAT | CTCGAG | ATGTCTCGCTTCTTCCTCTTC |  |
| STUB1-RNAi(32418-1)-a | Ccgg | cgCGAAGAAGAAGCGCTGGAA | CTCGAG | TTCCAGCGCTTCTTCTTCGCG | TTTTTg |
| STUB1-RNAi(32418-1)-b | GATCCAAAAA | cgCGAAGAAGAAGCGCTGGAA | CTCGAG | TTCCAGCGCTTCTTCTTCGCG |  |
| STUB1-RNAi(32419-1)-a | Ccgg | gcAGTCTGTGAAGGCGCACTT | CTCGAG | AAGTGCGCCTTCACAGACTGC | TTTTTg |
| STUB1-RNAi(32419-1)-b | GATCCAAAAA | gcAGTCTGTGAAGGCGCACTT | CTCGAG | AAGTGCGCCTTCACAGACTGC |  |
