# Supplementary material for: CHIP-mediated CIB1 ubiquitination regulated epithelial–mesenchymal transition and tumor metastasis in lung adenocarcinoma
Source: Cell Death Differ. 2020 Oct 20;28(3):1026–40. doi: 10.1038/s41418-020-00635-5 (PMC7937682; doi:10.1038/s41418-020-00635-5)
Supplement: Supplementary file 10 — Supplement Table 2 [file 41418_2020_635_MOESM10_ESM.docx]

| Primer Name | Primer sequence |
| --- | --- |
| CIB1 reverse | 5′-CTTGAGCTCTGGAAGGCTGA-3′ |
| CIB1 forward | 5′-AGTACCAGGACTTGACGTTCC-3′ |
| CHIP reverse | 5’ -TGCGTCAATAACCTCCTTCA-3′ |
| CHIP forward | 5’ - CTCCTACCTCTCCAGGCTCA-3’ |
| β-actin: reverse | 5’-TAGCACAGCCTGGATAGCAACGTA-3’ |
| β-actin: forward | 5’-ACCAACTGGGACGACATGGAGAAA-3’ |
| K10 reverse | 5′- CAGTCGCCTGTCCAGGGAGCTGCTG-3′ |
| K10 forward | 5′- CTGGACAGGCGACTGCCCGAGCCC-3′ |
| K24 reverse | 5′- GACGTTCCTGACGAGGCAGGAGATCC-3′ |
| K24 forward | 5′-CTCGTCAGGAACGTCAAGTCCTGGT-3′ |
| K65 reverse | 5′-CAGCCTTCCAGAGCTCAGGGCCAACCC-3′ |
| K65 forward | 5′-CTGAGCTCTGGAAGGCTGAGAATCTGCT-3′ |
| K70 reverse | 5′-AGGCCAACCCCTTCAGGGAGCGAATCT-3′ |
| K70 forward | 5′-CTGAAGGGGTTGGCCTTGAGCTCTGGA-3′ |
| K83 reverse | 5′-CTCCACATCCCCAGCCAGGGACAGCCTT-3′ |
| K83 forward | 5′-CCTGGCTGGGGATGTGGAGAAGACCCTGCA-3′ |
| K107 reverse | 5′-CAGCCACGCCAGACATCAGGTCCCATTAT-3′ |
| K107 forward | 5′-CTGATGTCTGGCGTGGCTGTGTCACT-3′ |
| K150 reverse | 5′-AGTGCGTCTGAGATGAGGCAGCTCATC-3′ |
| K150 forward | 5′-CTCATCTCAGACGCACTAAGCCGTGTGT-3′ |
| K188 reverse | 5′-TGCCAGCTCCTTTAGGATTGTCCTGT-3′ |
| K188 forward | 5′-CTAAAGGAGCTGGCAAAGTCTGGAGA-3′ |
