# Supplementary material for: CHIP-mediated CIB1 ubiquitination regulated epithelial–mesenchymal transition and tumor metastasis in lung adenocarcinoma
Source: Cell Death Differ. 2020 Oct 20;28(3):1026–40. doi: 10.1038/s41418-020-00635-5 (PMC7937682; doi:10.1038/s41418-020-00635-5)
Supplement: Supplementary file 11 — Supplement Table 3 [file 41418_2020_635_MOESM11_ESM.docx]

Supplement Table 3. CIB1 expression in lung adenocarcinoma tissues and matched normal lung tissues (n=90)

| Groups | Numbers | CIB1 Expression | | High Expression Rate（%） | χ^2^ | *P* Value |
| --- | --- | --- | --- | --- | --- | --- |
|  |  | Low | High |  |  |  |
| Lung Adenocarcinoma tissue | 60 | 24 | 36 | 60 | 10.91 | 0.001 |
| Normal Lung tissue | 60 | 42 | 18 | 30 |  |  |
