# Supplementary material for: CHIP-mediated CIB1 ubiquitination regulated epithelial–mesenchymal transition and tumor metastasis in lung adenocarcinoma
Source: Cell Death Differ. 2020 Oct 20;28(3):1026–40. doi: 10.1038/s41418-020-00635-5 (PMC7937682; doi:10.1038/s41418-020-00635-5)
Supplement: Supplementary file 12 — Supplement Table 4 [file 41418_2020_635_MOESM12_ESM.docx]

| Supplement Table 4. Proteins Identified In LS-MS | | | |
| --- | --- | --- | --- |
| Protein Name | UniProtKB AC | Peptide Sequence | Percent Coverage |
| YES | P07947 | EVLEQVER | 43 |
| ZSC29 | Q8IWY8 | LKPFQR | 28 |
| VAT1 | Q99536 | LPACVVDCGTGYTK | 151 |
| UE2NL | Q5JXB2 | TNEAQAIETAR | 58 |
| UBP4 | Q13107 | AAYVLFYQR | 24 |
| TMEDA | P49755 | LKPLEVELR | 27 |
| TTL12 | Q14166 | TELPQFVSYFQQR | 32 |
| STK35 | Q8TDR2 | LVETSLK | 16 |
| STAT3 | P40763 | KFNILGTNTK | 23 |
| SRSF9 | Q13242 | EAGDVCYADVQK | 21 |
| SGPL1 | O95470 | VAIQFLK | 17 |
| SI1L1 | O43166 | LSDFLITGGGK | 51 |
| SCN3A | Q9NY46 | GIDYVKNK | 13 |
| SCAM3 | O14828 | TAAANAAAGAAENAFR | 98 |
| SCMC1 | Q6NUK1 | SYWLDNFAK | 32 |
| RRAS2 | P62070 | QVTQEEGQQLAR | 38 |
| RORB | Q92753 | SGCLEVVLVRMCR | 20 |
| ROS1 | P08922 | LTLLVTR | 16 |
| REQU | Q92785 | GPGLASGQLYSYPAR | 39 |
| PIBF1 | Q8WXW3 | NQMALDLEQLLNHR | 18 |
| FLOT2 | Q9BTI6 | IGEAEAAVIEAMGK | 161 |
| PA2G4 | Q9UQ80 | TIIQNPTDQQK | 28 |
| PAI1 | P05121 | QVDFSEVER | 51 |
| NKRF | O15226 | EGLGLDVER | 20 |
| Rac3 | P60763 | LAPITYPQGLAMAR | 47 |
| NR4A3 | Q92570 | VLGALVELR | 17 |
| MK15 | Q8TD08 | TGEVVAIK 14 | 14 |
| MAGG1 | Q96MG7 | LVELEPK | 17 |
| ITB1 | P05556 | IGFGSFVEK | 23 |
| HCK | P08631 | VIEDNEYTAR | 27 |
| GNAI3 | P08754 | LFDSICNNK | 72 |
| SPHK1 | Q9NYA1 | YRRLGEMR | 43 |
| CUL1 | Q13616 | MFQDIGVSK | 25 |
| CHIP | Q9UNE7 | LNFGDDIPSALR | 64 |
| CA2D1 | P54289 | AKLEETITQAR | 72 |
| B2L12 | Q9HB09 | LASDPALR | 22 |
| AVL9 | Q8NBF6 | LILLEK | 21 |
| AIP | Q5VWQ8 | AVPLIHQEGNR | 42 |
