# Supplementary material for: CHIP-mediated CIB1 ubiquitination regulated epithelial–mesenchymal transition and tumor metastasis in lung adenocarcinoma
Source: Cell Death Differ. 2020 Oct 20;28(3):1026–40. doi: 10.1038/s41418-020-00635-5 (PMC7937682; doi:10.1038/s41418-020-00635-5)
Supplement: Supplementary file 13 — Supplement Table 5 [file 41418_2020_635_MOESM13_ESM.docx]

| Patients Characteristic | | | | | | | | | | | |
| --- | --- | --- | --- | --- | --- | --- | --- | --- | --- | --- | --- |
| Patient ID |  | Ages | Smoking History | CIB1 expression | T-stage | N-stage | M-stage | TNM-stage | OS | DFS | chemotherapy |
| 1 | Male | 55.00 | Non | Relatively low | 2.00 | 2.00 | 0.00 | 3.00 | 16 | 12 | YES |
| 2 | Female | 41.00 | Non | Relatively low | 3.00 | 2.00 | 0.00 | 3.00 | 24 | 20 | NON |
| 3 | Male | 59.00 | Non | Relatively low | 2.00 | 2.00 | 0.00 | 3.00 | 26 | 20 | NON |
| 4 | Female | 52.00 | Non | Relatively low | 1.00 | 0.00 | 0.00 | 1.00 | 30 | 27 | YES |
| 5 | Female | 60.00 | Non | Relatively low | 1.00 | 2.00 | 0.00 | 3.00 | 32 | 20 | YES |
| 6 | Male | 53.00 | Smoker | Relatively low | 2.00 | 1.00 | 0.00 | 2.00 | 32 | 28 | YES |
| 7 | Female | 60.00 | Smoker | Relatively low | 1.00 | 2.00 | 0.00 | 3.00 | 33 | 26 | NON |
| 8 | Male | 75.00 | Smoker | Relatively low | 3.00 | 0.00 | 0.00 | 2.00 | 38 | 24 | YES |
| 9 | Female | 60.00 | Smoker | Relatively low | 1.00 | 0.00 | 0.00 | 1.00 | 52 | 48 | NON |
| 10 | Male | 49.00 | Non | Relatively low | 1.00 | 0.00 | 0.00 | 1.00 | 52 | 48 | NON |
| 11 | Male | 74.00 | Smoker | Relatively low | 2.00 | 0.00 | 0.00 | 1.00 | 60 | 60 | NON |
| 12 | Male | 66.00 | Smoker | Relatively low | 2.00 | 0.00 | 0.00 | 2.00 | 60 | 60 | YES |
| 13 | Male | 61.00 | Smoker | Relatively low | 1.00 | 0.00 | 0.00 | 1.00 | 60 | 60 | NON |
| 14 | Female | 60.00 | Non | Relatively low | 1.00 | 0.00 | 0.00 | 1.00 | 60 | 60 | YES |
| 15 | Female | 58.00 | Smoker | Relatively low | 1.00 | 0.00 | 0.00 | 1.00 | 60 | 60 | YES |
| 16 | Female | 58.00 | Non | Relatively low | 1.00 | 0.00 | 0.00 | 1.00 | 60 | 60 | NON |
| 17 | Female | 52.00 | Non | Relatively low | 1.00 | 0.00 | 0.00 | 1.00 | 60 | 60 | YES |
| 18 | Male | 49.00 | Smoker | Relatively low | 2.00 | 0.00 | 0.00 | 2.00 | 60 | 60 | NON |
| 19 | Female | 49.00 | Non | Relatively low | 1.00 | 0.00 | 0.00 | 1.00 | 60 | 60 | YES |
| 20 | Male | 49.00 | Smoker | Relatively low | 2.00 | 0.00 | 0.00 | 1.00 | 60 | 60 | YES |
| 21 | Female | 46.00 | Non | Relatively low | 1.00 | 0.00 | 0.00 | 1.00 | 60 | 60 | NON |
| 22 | Male | 45.00 | Non | Relatively low | 1.00 | 0.00 | 0.00 | 1.00 | 60 | 60 | NON |
| 23 | Male | 66.00 | Smoker | Relatively low | 3.00 | 0.00 | 0.00 | 2.00 | 60 | 60 | NON |
| 24 | Male | 52.00 | Non | Relatively low | 3.00 | 0.00 | 0.00 | 2.00 | 60 | 60 | YES |
| 25 | Male | 62.00 | Smoker | Relatively High | 2.00 | 2.00 | 0.00 | 3.00 | 9 | 6 | YES |
| 26 | Female | 48.00 | Non | Relatively High | 1.00 | 1.00 | 0.00 | 2.00 | 12 | 8 | NON |
| 27 | Male | 43.00 | Smoker | Relatively High | 3.00 | 2.00 | 0.00 | 3.00 | 12 | 9 | NON |
| 28 | Female | 49.00 | Non | Relatively High | 2.00 | 2.00 | 0.00 | 2.00 | 12 | 9 | NON |
| 29 | Female | 48.00 | Non | Relatively High | 1.00 | 1.00 | 0.00 | 2.00 | 15 | 12 | YES |
| 30 | Male | 70.00 | Smoker | Relatively High | 3.00 | 2.00 | 0.00 | 3.00 | 18 | 16 | YES |
| 31 | Male | 74.00 | Smoker | Relatively High | 1.00 | 2.00 | 0.00 | 3.00 | 18 | 20 | YES |
| 32 | Female | 62.00 | Smoker | Relatively High | 2.00 | 2.00 | 0.00 | 3.00 | 21 | 14 | YES |
| 33 | Female | 60.00 | Non | Relatively High | 2.00 | 0.00 | 0.00 | 1.00 | 22 | 13 | YES |
| 34 | Female | 42.00 | Non | Relatively High | 1.00 | 2.00 | 0.00 | 3.00 | 22 | 15 | NON |
| 35 | Female | 49.00 | Non | Relatively High | 1.00 | 1.00 | 0.00 | 2.00 | 22 | 16 | NON |
| 36 | Female | 52.00 | Smoker | Relatively High | 1.00 | 1.00 | 0.00 | 2.00 | 26 | 22 | NON |
| 37 | Female | 58.00 | Non | Relatively High | 3.00 | 0.00 | 0.00 | 2.00 | 27 | 20 | YES |
| 38 | Female | 73.00 | Non | Relatively High | 1.00 | 2.00 | 0.00 | 3.00 | 28 | 22 | NON |
| 39 | Female | 52.00 | Non | Relatively High | 1.00 | 1.00 | 0.00 | 2.00 | 29 | 22 | YES |
| 40 | Male | 44.00 | Smoker | Relatively High | 3.00 | 2.00 | 0.00 | 3.00 | 29 | 24 | YES |
| 41 | Male | 52.00 | Smoker | Relatively High | 3.00 | 0.00 | 0.00 | 2.00 | 30 | 22 | YES |
| 42 | Female | 43.00 | Non | Relatively High | 3.00 | 1.00 | 0.00 | 3.00 | 32 | 20 | NON |
| 43 | Male | 49.00 | Non | Relatively High | 1.00 | 2.00 | 0.00 | 3.00 | 32 | 26 | YES |
| 44 | Male | 77.00 | Smoker | Relatively High | 2.00 | 2.00 | 0.00 | 3.00 | 32 | 30 | NON |
| 45 | Male | 63.00 | Smoker | Relatively High | 3.00 | 2.00 | 0.00 | 1.00 | 34 | 25 | YES |
| 46 | Male | 72.00 | Non | Relatively High | 3.00 | 0.00 | 0.00 | 2.00 | 36 | 24 | YES |
| 47 | Male | 53.00 | Smoker | Relatively High | 1.00 | 0.00 | 0.00 | 1.00 | 36 | 29 | YES |
| 48 | Female | 65.00 | Non | Relatively High | 2.00 | 0.00 | 0.00 | 2.00 | 36 | 31 | NON |
| 49 | Female | 67.00 | Non | Relatively High | 2.00 | 1.00 | 0.00 | 1.00 | 38 | 34 | NON |
| 50 | Male | 62.00 | Smoker | Relatively High | 2.00 | 0.00 | 0.00 | 1.00 | 42 | 32 | YES |
| 51 | Male | 66.00 | Non | Relatively High | 2.00 | 0.00 | 0.00 | 1.00 | 53 | 47 | NON |
| 52 | Male | 54.00 | Smoker | Relatively High | 1.00 | 0.00 | 0.00 | 1.00 | 55 | 49 | NON |
| 53 | Male | 59.00 | Non | Relatively High | 1.00 | 0.00 | 0.00 | 1.00 | 60 | 60 | YES |
| 54 | Male | 55.00 | Smoker | Relatively High | 1.00 | 0.00 | 0.00 | 1.00 | 60 | 60 | YES |
| 55 | Male | 53.00 | Smoker | Relatively High | 1.00 | 0.00 | 0.00 | 1.00 | 60 | 60 | NON |
| 56 | Female | 48.00 | Non | Relatively High | 1.00 | 0.00 | 0.00 | 1.00 | 60 | 60 | NON |
| 57 | Male | 48.00 | Non | Relatively High | 2.00 | 0.00 | 0.00 | 2.00 | 60 | 60 | NON |
| 58 | Male | 47.00 | Smoker | Relatively High | 1.00 | 0.00 | 0.00 | 1.00 | 60 | 60 | NON |
| 59 | Female | 67.00 | Smoker | Relatively High | 1.00 | 1.00 | 0.00 | 2.00 | 60 | 60 | NON |
| 60 | Male | 54.00 | Smoker | Relatively High | 2.00 | 1.00 | 0.00 | 2.00 | 60 | 60 | YES |
